# Does shift-and-persist strategy buffer career choice anxiety and affect career exploration?

**DOI:** 10.1186/s13104-022-06206-w

**Published:** 2022-09-24

**Authors:** Sumin Lee, Ryota Kobayashi, Mami Oda, Yoshihide Noritake, Ken’ichiro Nakashima

**Affiliations:** 1 Graduate School of Education, Hiroshima University, 1-1-1 Kagamiyama, Higashi-Hiroshima City, Hiroshima 739-8524 Japan; 2 Society for the Promotion of Science, Tokyo, Japan; 3 Faculty of Human and Social Sciences, Fukuoka Prefectural University, Fukuoka, Japan; 4 Graduate School of Education, Hiroshima University, Higashi-Hiroshima, Japan; 5 Kawasaki University of Medical Welfare, Kurashiki, Japan; 6 Department of Psychology, Graduate School of Humanities and Social Sciences, Hiroshima University, Higashi-Hiroshima, Japan

**Keywords:** Shift-and-persist strategy, Socioeconomic status, Career, Psychological health

## Abstract

**Objective:**

The transition from school to the workforce is important for concrete future planning. During this period, people are more likely to experience psychological health problems, such as anxiety and feelings of hopelessness and helplessness. In particular, job hunting in individuals with low socioeconomic status (SES) leads to various impulsive behaviors and physical and psychological problems due to a scarcity of economic and time resources. There is a lack of research examining career education and intervention approaches that consider the backgrounds of those experiencing adversities and difficulties due to low SES. Considering these situations, we examined whether shift-and-persist coping strategies (S-P) could buffer the career choice anxiety of individuals with low SES and improve career exploration.

**Results:**

The results from 311 students who preparing/doing for job hunting showed a negative association between S-P and career choice anxiety and a positive association with career exploration. There are no significant effects of the direct link between SES to career exploration and the indirect link between SES and career exploration via career choice anxiety. There was also no buffering effect of S-P use on the above mediating process.

## Introduction

A high level of anxiety has a disturbing effect on job hunting [1]; it can also be a direct or indirect risk factor that hinders career exploration. Low socioeconomic status (SES), an individual difference factor, is detrimental to individuals’ physical and psychological health [2] and their job hunting anxiety and career-related indicators. Low SES individuals tend to stop career exploration because of a scarcity of external resources (e.g., time and money) [3]. However, no previous study has considered SES or revealed its association with job hunting anxiety and career-related indicators.

Although recent approaches have been taken to mitigate anxiety about job hunting [4], blindly introducing and implementing career education without understanding an individual's situation and context can lead to confusion in job hunting activities of low SES. They can lead to undesirable outcomes, such as taking up a job that is inconsistent with one's intentions and aptitude.

**Fig. 1:**
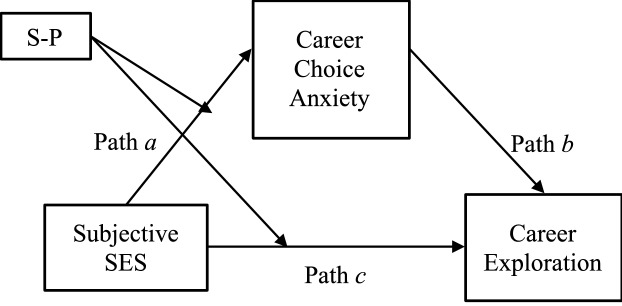
Overview of the moderated mediation analysis

Hence, we focused on the shift-and-persist strategy (S-P; [5, 6]), which is considered an effective coping strategy for protecting psychological health and reducing risky and impulsive decision-making of low SES individuals. Furthermore, we addressed a moderated mediation model in which SES led to career choice anxiety and influenced career exploration, as in Fig. 1. Specifically, we examined the direct effect of SES on career exploration and the indirect effect of career choice anxiety as mediating variables. We also investigated whether low SES individuals with higher S-P scores had lower career choice anxiety and were more active in career exploration.

## Main text

### Method

#### Participants and procedures

We recruited and conducted an online survey among 395 monitors registered with the crowdsourcing company in Japan (CrowdWorks; https://crowdworks.jp/static/lp/research/). The questionnaire was created using Google Forms, and participants were asked to complete it online using their own PCs or smartphones. We excluded participants who met the following criteria: (1) were not students at vocational, junior college, university, or graduate school (*N* = 7), (2) did not meet the age requirement of being between 18 and 29 years old (*N* = 14), (3) responded to the filler item—‘‘Be sure to choose not to answer this question’’—from the analysis (*N* = 42), and (4) met all of (1) to (3) criteria (*N* = 21). Finally, 311 participants (122 males, 182 females, and 7 others; *M*_*age*_ = 22.68, *SD* = 2.99) were included in the analysis. The study was conducted from mid-to-late January 2021 with the approval of the Research Ethics Committee of Hiroshima University.

### Scales

SES was measured using an established three-item measure from [7] the frequency with which participants experienced a lack of resources in their childhood living environment (up to approximately age 12) using the Likert scale (from 1 ‘‘strongly disagree’’ to 7 ‘‘strongly agree’’). An example is ‘‘My family usually had enough money for things when I was growing up”.

The S-P combines two coping strategies: *Shifting* and *Persisting*. First, *shifting* involves the acceptance and positive reframing of the negative aspects of adversity, stressful events, and practicing emotional regulation. Second, *persisting* involves finding meaning in one’s life and circumstances and maintaining an optimistic outlook about the future. S-P was measured using four established shifting items such as ‘‘I think about what I can learn from the situation,” three persisting items such as “I feel my life has a sense of purpose,” and six distractor measures from [8, 9], using a Likert scale (from 1 “disagree” to 4 ‘‘agree’’).

Career choice anxiety was assessed using established sixteen-item measures from short forms of [10], on four subscales of transition to work anxiety, self-awareness anxiety, decision strategy anxiety, and career-awareness anxiety, using the Likert scale (from 1 ‘‘strongly disagree ‘‘to 5’’strongly agree”). Examples are ‘‘I am worried about my ability to be independent as a member of society,” ‘‘I am worried that I do not know which profession or job is interesting,” ‘‘It is difficult to narrow down the many occupations and jobs to one,” ‘‘I am worried that I do not know the contents of the occupation and job’’.

Career exploration was assessed using established thirteen-item measures from [11], on two subscales of environmental exploration and self-exploration, participants were answered using Likert scale (from 1 ‘‘not at all‘‘ to 5 ”very often’’). Examples are "Read books, magazines, or internet articles related to jobs and work,” ‘‘Thought about my favorite things and strong points,” etc.

### Data Analysis

We analyzed using statistical software HAD 17_204 [12] and *M*plus version 8.5 [13]. We computed individual averaging scores all scales except for S-P. We made S-P scores by creating mean scores for shifting and persisting separately, standardizing these mean scores, and then averaging these standardized scores [14]. All scores indicated that higher composite scores indicated greater subjective childhood SES, S-P, career choice anxiety, and career exploration. Before conducting the moderated mediation analysis, the following steps were performed (cf., [15]): (1) The direct effect of SES on career exploration and the indirect effects via career choice anxiety were examined by performing the mediation analysis; (2) Through the moderation analysis was examined whether S-P can buffer the association between SES and career choice anxiety, and between SES and career exploration. In addition, 95% confidence intervals were reported in this analysis. The effects were present when the 95% confidence interval did not cross zero when interpreting the results. Gender and age were also included as covariates in conducting the moderated mediation analysis.

### Results

Table 1 shows the scale’s descriptive statistics, reliability coefficients, and correlation coefficients.

**Table 1:** Descriptive statistics, reliability coefficients, and correlation coefficients between scales

	*α*	*Total score*	*SD*	1		2		3		4
1 Socioeconomic status	0.79	12.48	4.07	–						
2 Shift-and-persist strategy	0.83	0.04^a^	0.86	0.15	^**^	–				
3 Career choice anxiety	0.95	55.18	15.90	0.04		− 0.28	^***^	–		
4 Career exploration	0.83	44.57	7.74	0.04		0.45	^***^	− 0.17	^**^	–

^a^The original total S-P score was 0.00 (more precisely, 0.00038), 0.00038 was multiplied by 100 and shown to two decimal places to avoid misunderstanding

^**^p < 0.01

^***^p < 0.001

We first performed a mediation analysis to examine the process by which SES affected career exploration via career choice anxiety. The direct effect of SES on career choice anxiety was *b* = 0.14 (95%CI_bs_ [− 0.29, 0.59], β = 0.04), and the direct effect of SES on career exploration was *b* = 0.11 (95%CI_bs_ [− 0.10, 0.32], β = 0.06). The direct effect of career choice anxiety on career exploration was *b* = − 0.08 (95%CI_bs_ [− 0.14, − 0.02], β = − 0.17). We examined the mediation effect using bootstrapping (sample size = 5,000, same as below) and found that the mediation effect was *b* = − 0.01 (95%CI_bs_ [− 0.06, 0.02], β = − 0.02).

Second, we performed a moderation analysis to determine whether S-P can buffer the association between SES and career choice anxiety and between SES and career exploration. The interaction between SES and S-P on career choice anxiety was not found *b* = 0.23 (95%CIbs [− 0.31, 079], β = 0.16). Only the main effect of S-P was observed; *b* = − 7.63 (95%CIbs [− 14.20, − 1.30], β = − 0.41). Similarly, no interaction between SES and S-P on career exploration was found *b* = − 0.00 (95%CIbs [− 0.24, 0.24], β = − 0.01); only the main effect of S-P was observed *b* = 4.11 (95%CIbs [1.18, 6.95], β = 0.46).

Although no moderation effect was found in previous analyses, to test the main hypothesis model (Fig. 1), moderated mediation analysis with career choice anxiety as the mediator and S-P as the moderator was performed. The results showed no direct effects of career exploration from SES or indirect effects of career choice anxiety. There was also no moderating effect of S-P on the association between SES and career choice anxiety or career exploration (Table 2).

**Table 2:** The results of moderated mediation analysis in career choice anxiety and career exploration

Paths	*b* [95% CI], β
Direct effects	
SES → career exploration	− 0.02 [− 0.21, 0.17], − 0.01
Career choice anxiety → career exploration	− 0.03 [− 0.08, 0.03], − 0.05
S-P → career exploration	**3.83 [0.92, 6.70], 0.43**
SES → career choice anxiety	0.32 [− 0.13, 0.75], 0.08
S-P → career choice anxiety	− **7.62 [**− **14.22, **− **1.40], **− **0.41**
Interaction effects	
SES ×S-P → career exploration	0.07 [− 0.23, 0.24], 0.01
SES ×S-P → career choice anxiety	0.22 [− 0.30, 0.79], 0.15
Conditional indirect effect	
SES ×lower S-P → career choice anxiety → career exploration	− 0.00 [− 0.05, 0.01], − 0.00
SES ×higher S-P → career choice anxiety → career exploration	-0.01 [− 0.08, 0.01], − 0.01

Number of bootstrap samples for percentile bootstrap confidence intervals: 5000. *b* = unstandardized coefficient, β = standardized beta, CI = confidence interval, SES = socioeconomic status, S-P = shift-and-persist strategy. Significant paths are shown in bold

The results of the S-P did not moderate the associations with SES, career choice anxiety, and career exploration. Nevertheless, the moderation analysis suggests that the observed buffering effects of S-P on career choice anxiety and career exploration show that S-P may reduce career choice anxiety and promote career exploration. Furthermore, the mediation analysis found a path of career choice anxiety to career exploration, indicating that decreased career anxiety is associated with improved career exploration.

## Discussion

Our data suggested that regardless of higher or lower SES, using more S-P buffered career choice anxiety and performed more career exploration. Career choice anxiety negatively affected career exploration. However, because a direct link between SES and career choice anxiety or career exploration was not shown, our data are insufficient to argue the need to consider the context of SES when providing career education and intervention. Although our model was not supported, our results suggest that a cognitive/thinking style of acceptance and positive reframing of stressful events, finding meaning in life, and optimism about the future (i.e., S-P) may reduce people to anxiety in job hunting and promote career exploration. Moreover, the negative association between career choice anxiety and career exploration is evidence that supports previous studies that have addressed anxiety as a psychological problem in job hunting and have shown a negative association with maladjustments in job hunting, such as job hunting activities [1] and career decisions [16].

In addition, since this association disappeared in the moderated mediation analysis model (β = − 0.05, *n.s.*), we can assume that in this analysis, S-P controlled for emotional regulation difficulties and dysregulation aspects of the Career Choice Anxiety scale and offset the relationship between career choice anxiety and career exploration. Indeed, since the Career Choice Anxiety scale has items on emotional regulation difficulties and dysregulation, it is likely that individual differences in emotional dysregulation, which are different from basic anxiety, were negatively related to career exploration. Furthermore, this can also be considered evidence supporting that the S-P strategy works against the dysregulation aspects of career choice anxiety and is effective in breaking the negative association between career choice anxiety and career exploration. However, the role of S-P in psychological health is less clear, and the results are controversial. Future studies will need to build on the empirical evidence examining the effects of S-P on psychological health. Despite some limitations as noted bellow, the current study demonstrates the necessity for S-P education and interventions for anxiety in all students doing and preparing for job hunting; extends the impact of anxiety on career exploration; and provides initial insights into its psychological processes.

## Limitations

First, we only used measures of anxiety in general career choices. Individuals with low SES have less time and financial resources for job hunting [3] and have difficulty accessing external resources [17], and it is possible that the type of anxiety felt by low SES individuals (e.g., anxiety over transportation costs) may differ. Future research is necessary to examine individuals with low SES anxiety by adding more accurate and measurable indicators to provide systematic and specific education and intervention methods related to career choice anxiety. Second, we focused only on the subjective childhood SES. In fact, we also collected the current objective SES (e.g., family income), but due to a lack of responses, we could not use this variable in our analysis. As noted above, those with low SES may more likely be exposed to current times, financial constraints, and threats in their job hunting. Therefore, future studies should be creative in measuring current SES, such as asking about the social and economic difficulties they face during their job hunting or asking about family income and educational attainment. Relatedly, in terms of person-by-context models of emotional regulation, it has been demonstrated that coping strategies such as reappraisal are more adaptive in contexts with limited control opportunities [18, 19]. Heckhausen et al. [20] demonstrated that reappraisal is equally helpful for health outcomes when individuals from higher SES backgrounds face uncontrollable stressors (such as chronic illness). Future consideration must be given to the applicability of S-P, which must include not only SES but also contextual and environmental controllability. Finally, although the current study was conducted in technical schools, junior colleges, universities, and graduate students, we could not examine differences by participant characteristics. The degree of association between the variables in the study may differ by major, and academic background, and there is room for discussion in future studies.

## Data Availability

The dataset supporting the conclusions of this article is available in the Open Science Framework, hyperlink to dataset(s) in https://osf.io/qvc3b/?view_only=9dce7a121e37482eb06d91323d5e9b06.
